# Renal Effects and Nitric Oxide Response Induced by *Bothrops atrox* Snake Venom in an Isolated Perfused Kidney Model

**DOI:** 10.3390/toxins17080363

**Published:** 2025-07-24

**Authors:** Terentia Batista Sa Norões, Antonio Rafael Coelho Jorge, Helena Serra Azul Monteiro, Ricardo Parente Garcia Vieira, Breno De Sá Barreto Macêdo

**Affiliations:** 1 Department of Medicine, Centro Universitário Doutor Leão Sampaio (UNILEÃO), Maria Letícia Leite Pereira Ave., Lagoa Seca—Cidade Universitária, Juazeiro do Norte 63040-405, CE, Brazil; brenodesamacedo@gmail.com; 2 Department of Physiology and Pharmacology, School of Medicine, Federal University of Ceara, Coronel Nunes de Melo St., 1127, Fortaleza 60430-275, CE, Brazil; antoniorafaelj@yahoo.com.br (A.R.C.J.); hsazul@gmail.com (H.S.A.M.); 3 Department of Medicine, Federal University of Cariri (UFCA), Ten. Raimundo Rocha Ave., 1639, Juazeiro do Norte 63048-080, CE, Brazil; ricardopgv@gmail.com

**Keywords:** *Bothrops atrox*, renal perfusion, oxide nitric

## Abstract

The snakes from the genus *Bothrops* are responsible for most of the ophidic accidents in Brazil, and *Bothrops atrox* represents one of these species. Envenomation by these snakes results in systemic effects and is often associated with early mortality following snakebite incidents. The present study investigates the pharmacological properties of *Bothrops atrox* venom (VBA), focusing specifically on its impact on renal blood flow. Following the renal perfusion procedure, kidney tissues were processed for histopathological examination. Statistical analysis of all evaluated parameters was conducted using ANOVA and Student’s *t*-test, with significance set at *p* < 0.005. Administration of VBA resulted in a marked reduction in both perfusion pressure and renal vascular resistance. In contrast, there was a significant elevation in urinary output and glomerular filtration rate. Histological changes observed in the perfused kidneys were mild. The involvement of nitric oxide in the pressor effects of *Bothrops atrox* venom was not investigated in renal perfusion systems or in in vivo models. Treatment with VBA led to elevated nitrite levels in the bloodstream of the experimental animals. This effect was completely inhibited following pharmacological blockade with L-NAME. Based on these findings, we conclude that VBA alters renal function and promotes increased nitric oxide production.

## 1. Introduction

Snakebite envenomation represents a serious public health concern in tropical countries. According to the World Health Organization, snakebites affect between 4.5 and 5.4 million people each year, resulting in up to 138,000 deaths annually [1]. In Brazil, more than 32,000 snakebite cases are registered annually. These data highlight the importance of effective preventive measures and treatments to mitigate the impact of envenomation [2,3]. A more recent study conducted in the state of Ceará between 2019 and 2022 reported 3102 cases of snakebite envenomation, resulting in 14 deaths [4,5]. The majority of incidents were caused by snakes of the genus *Bothrops*, with most victims being males aged 20 to 59 years.

*Bothrops atrox* is the most medically important snake in the Amazon region, accounting for the majority of envenomation cases not only in Brazil but also in neighboring countries such as Ecuador, Peru, Colombia, Venezuela, and Bolivia. Its wide geographic distribution and high incidence of bites highlight the critical need for research into its venom’s pathophysiological effects and potential therapeutic interventions [6].

Envenomation by the *Bothrops* species commonly leads to tissue injury characterized by edema, necrosis, and the formation of abscesses, effects primarily attributed to the action of proteolytic enzymes present in the venom [7]. Hypotension has been frequently reported in *Bothrops* envenomation and represents a systemic effect that may lead to inadequate perfusion of multiple organs. In such conditions, a reduction in renal intraglomerular pressure may occur, potentially leading to cardiac failure or hypoxia [7]. Among fatal snakebite cases in Brazil, acute kidney injury (AKI) is the most common complication and the leading cause of death following *Bothrops* envenomation [8,9].

Snake venoms are known for their biochemical complexity, containing over 20 different components, with proteins accounting for approximately 90% of the dry weight. *Bothrops atrox* venom is composed of a complex mixture of biologically active molecules, including metalloproteinases (SVMPs), serine proteases (SVSPs), phospholipases A_2_ (PLA_2_), L-amino acid oxidases (LAAO), C-type lectins, disintegrins, and bradykinin-potentiating peptides [10,11]. The venom of *Bothrops atrox* is known to induce a range of pathophysiological effects, including hemorrhage, renal dysfunction, and alterations in vascular integrity, which contribute significantly to systemic toxicity and organ damage [12,13].

Besides venom components, other substances also contribute to envenomation pathophysiology, including the release of various endogenous mediators, which further complicates the systemic effects of snakebites. Histamine, bradykinin, eicosanoids, platelet-activating factor (PAF), and autonomic neurotransmitters have all been proposed as participants in the systemic manifestations induced by *Bothrops* envenomation [14,15,16,17].

Snakebite envenomation remains a significant public health concern in Brazil, particularly due to its potential to cause renal complications. Although previous studies using experimental models have shown that *Bothrops* venom can severely impair renal function [18], the precise mechanisms responsible for this nephrotoxicity are not yet fully understood. Understanding these mechanisms is crucial not only for developing targeted therapies for envenomated patients but also for identifying novel bioactive compounds with potential pharmacological applications. In this context, the present study offers original insights into the renal effects of crude *Bothrops atrox* venom and contributes to the broader understanding of venom-induced renal pathophysiology.

## 2. Results

**Figure 1:**
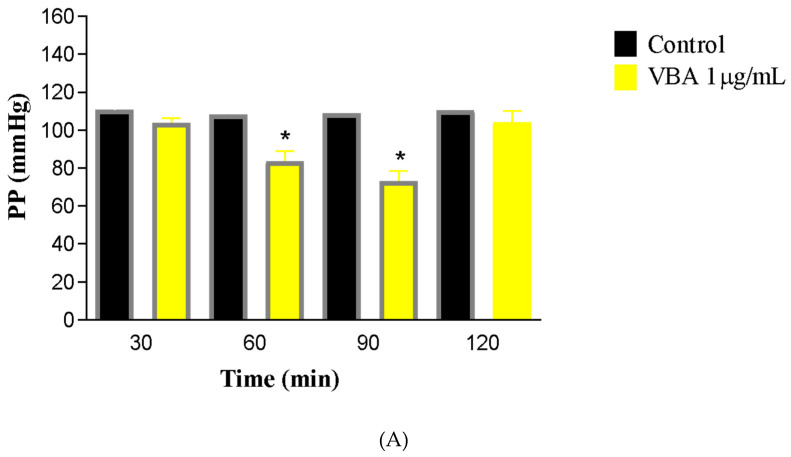

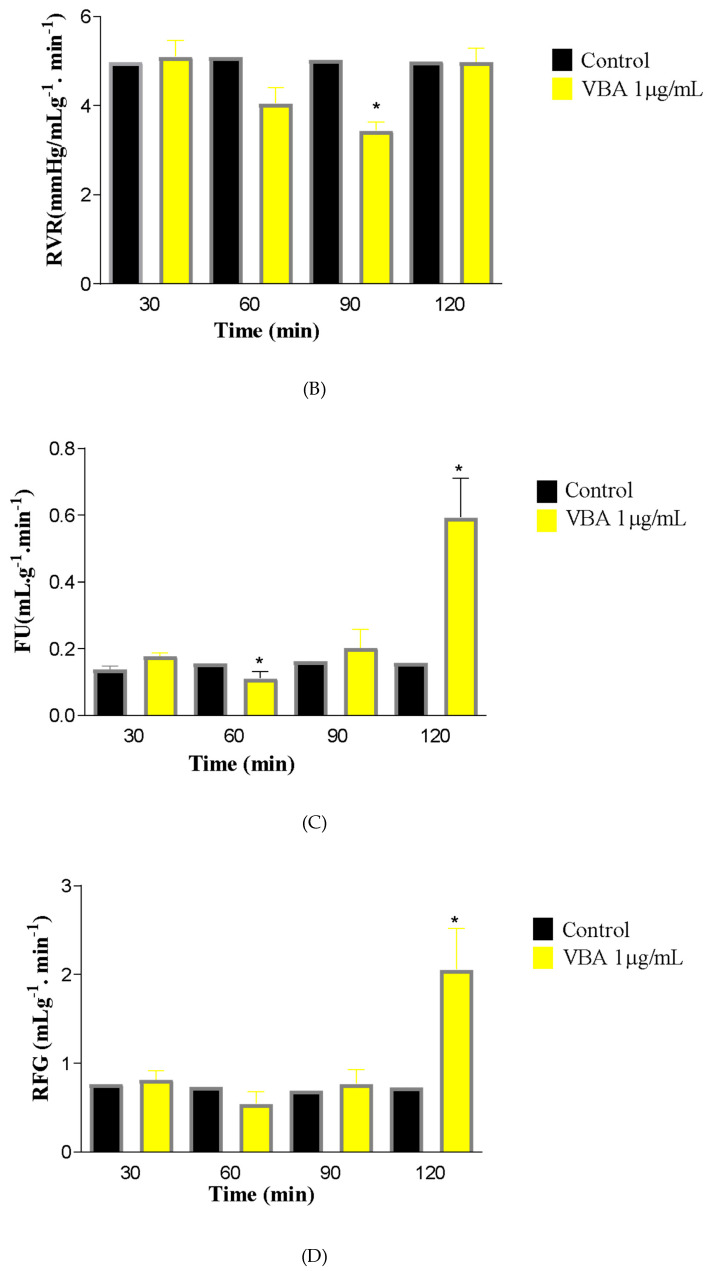

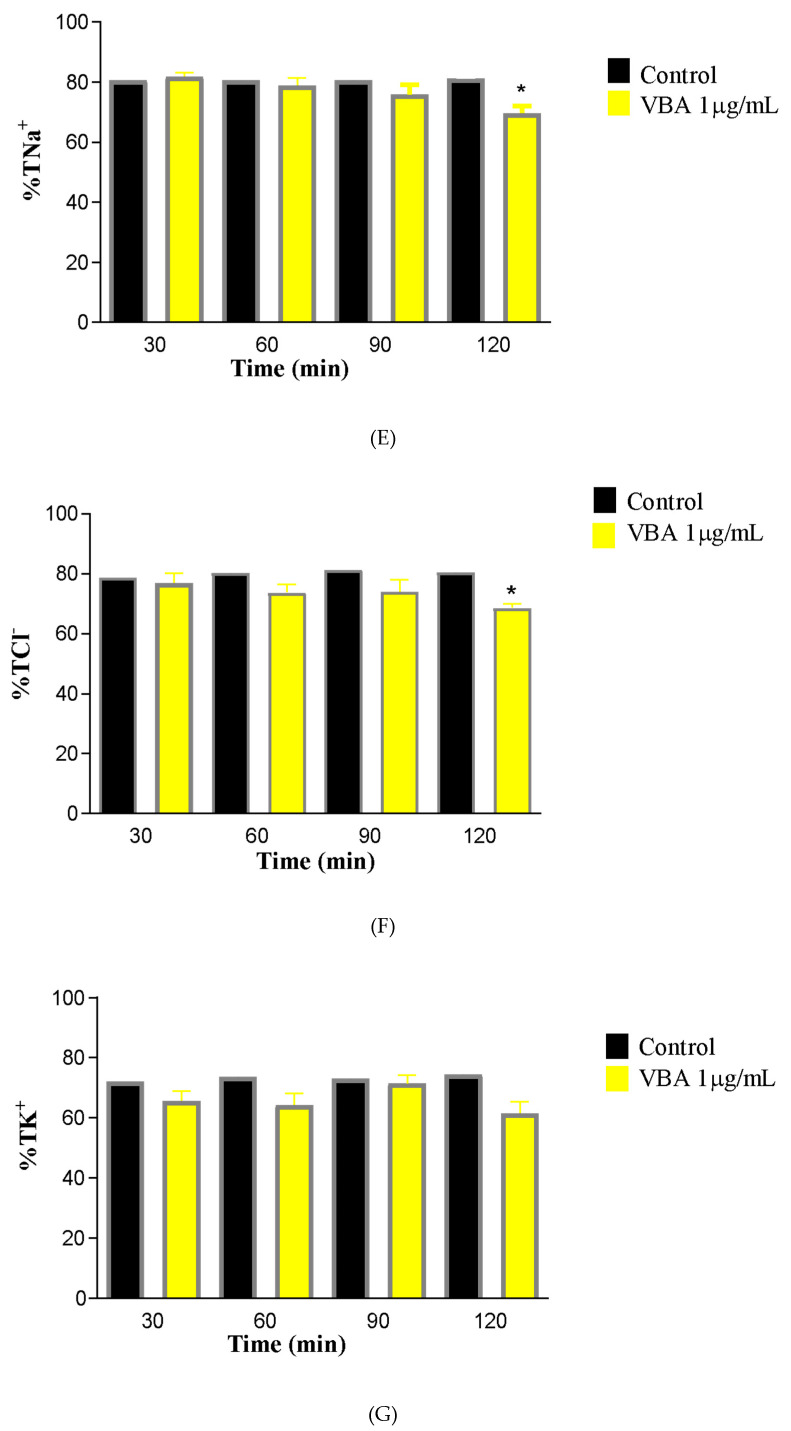

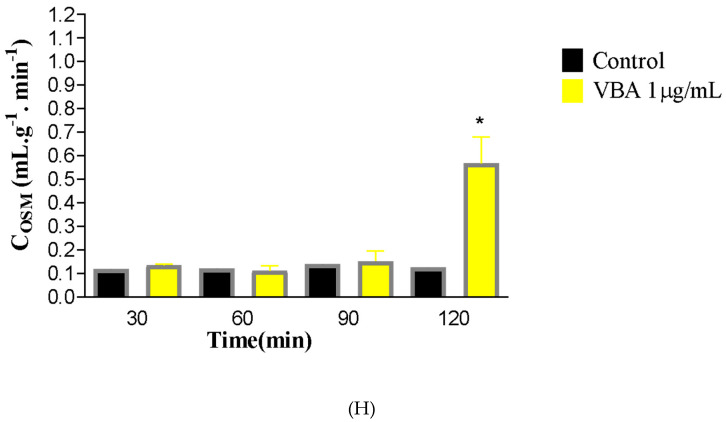
Impact of *Bothrops atrox* venom (VBA, 1.0 μg/mL) on the following renal parameters: perfusion pressure (PP; **A**), renal vascular resistance (RVR; **B**), urinary flow (UF; **C**), glomerular filtration rate (GFR; **D**), sodium tubular transport (%TNa^+^; **E**), potassium tubular transport (%TCl^−^; **F**), chloride tubular transport (%TK^+^; **G**); and osmotic clearance (COSM; **H**). Data are presented as mean ± SEM from six kidneys per group. Statistical comparisons were made between time points (60, 90, and 120 min) and the baseline control at 30 min using one-way ANOVA followed by Student’s *t*-test, considering * *p* < 0.05 as statistically significant.

The introduction of crude *Bothrops atrox* venom at a concentration of 1 μg/mL into the rat renal perfusion system led to a decline in vascular parameters beginning at 60 min, with a more substantial reduction noted at the 90-min mark (Figure 1). Perfusion pressure (PP) decreased to 73.01 ± 5.5 mmHg compared to the control value of 103.6 ± 2.5 mmHg. Renal vascular resistance (RVR) showed a 21% reduction (C30 = 5.101 ± 0.37; BaV90 = 4.052 ± 0.35 mmHg/mL/g/min).

Functional parameters were also altered. From 60 min of perfusion onward, urinary flow (UF) was reduced; however, a reversal occurred, and by 120 min, a significant increase was observed, reaching values of 0.5937 ± 0.12 mL/g/min, equivalent to 71% of the control (C30 = 0.1785 ± 0.009). Glomerular filtration rate (GFR) increased by 60% compared to control values (C30 = 0.8163 ± 0.10; BaV120 = 2.059 ± 0.4631 mL/g/min).

Alterations were also observed in electrolyte handling. A decrease in sodium tubular transport (%TNa^+^) was detected at the 120-min point of the perfusion protocol (C30 = 81.76 ± 1.46; BaV120 = 69.63 ± 2.24). A reduction in chloride tubular transport (%TCl^−^) was also noted at 120 min (C30 = 76.80 ± 3.34; BaV120 = 68.55 ± 1.51). Conversely, potassium transport (%TK^+^) remained unchanged, with no statistically significant differences observed.

To evaluate the ability of the isolated kidney to clear osmotically active molecules, osmotic clearance (COSM) was calculated. BaV induced a significant increase in this parameter at 120 min compared to the control group (C30 = 0.1340 ± 0.008; BaV120 = 0.5673 ± 0.112).

**Figure 2:**
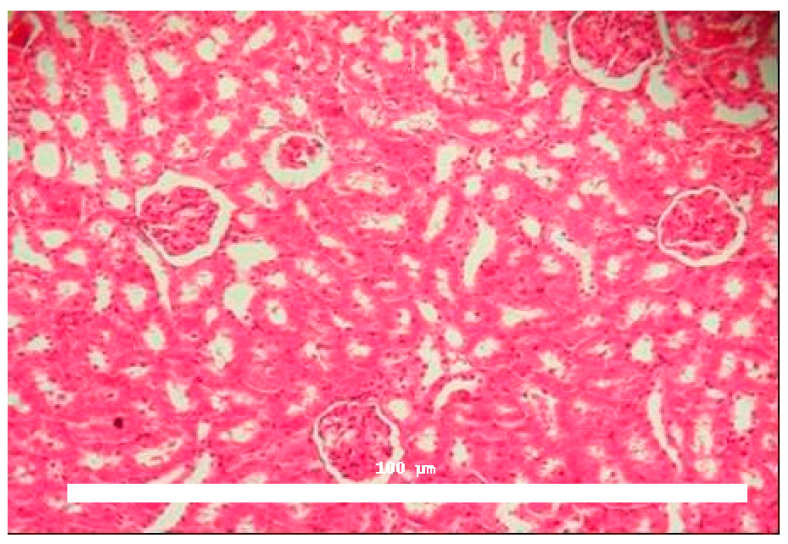
Histological evaluation of kidneys perfused with Krebs–Henseleit solution. No morphological alterations were identified in glomerular or tubular structures, and medullary tubules appeared normal in control samples. Magnification: 100×, hematoxylin and eosin staining.

**Figure 3:**
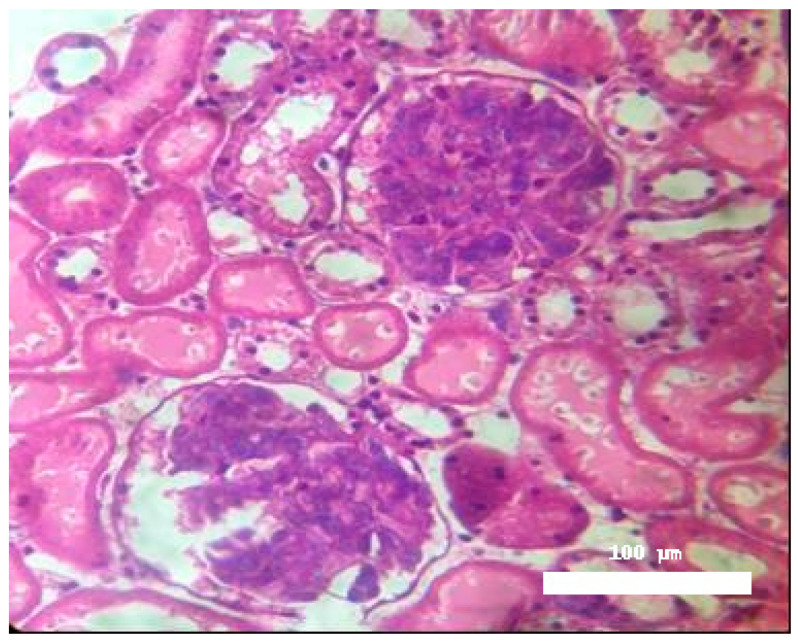
Histological examination of kidneys perfused with *Bothrops atrox* venom (VBA, 1.0 μg/mL) revealed the presence of intratubular proteinaceous deposits. Magnification: 400×, stained with hematoxylin and eosin.

Significant morphological alterations were also observed. Figure 2 shows a histological section of a kidney perfused solely with Krebs–Henseleit solution, demonstrating no structural changes induced by the vehicle. The examined slides showed that glomeruli, tubules, vessels, and interstitial tissue appeared normal. In contrast, kidneys perfused with BaV displayed tubules containing a substantial amount of proteinaceous material (Figure 3).

**Figure 4:**
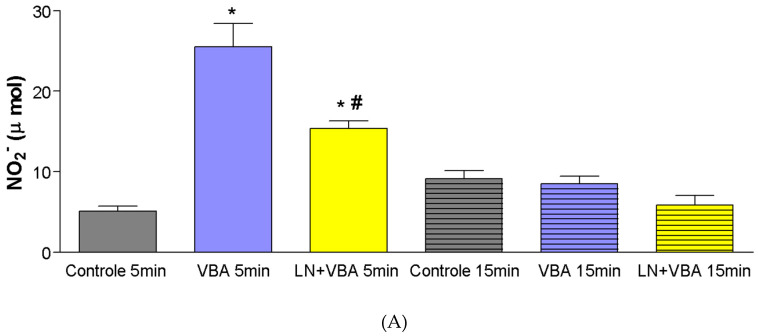

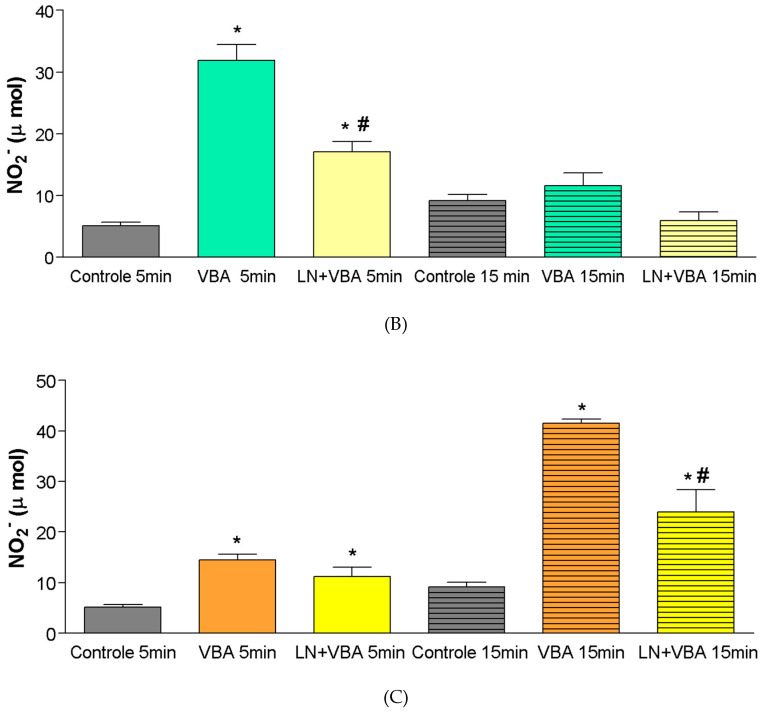
Nitrite concentrations measured in experimental groups following administration of crude *Bothrops atrox* venom (BaV) at a dose of 0.056mg/kg (**A**), 0.168mg/kg (**B**) and 0.28 mg/kg (**C**). Assessments were performed at 5 and 15 min post-infusion. Statistical analysis included one-way ANOVA with Newman–Keuls post hoc test and Student’s *t*-test, considering * *p* < 0.05 as statistically significant and *# *p* < 0.05, significantly different from the respective BaV-treated group. Results are shown as mean ± SEM, compared to control and BaV-treated groups at each respective time point.

A significant increase in nitrite production (µmol) was observed following administration of doses of *Bothrops atrox* venom (BaV), five minutes after infusion (Control_5_min = ±5 µmol; BaV 0.056 mg/kg_5_min = ±26 µmol; BaV 0.168 mg/kg_5_min = ±32 µmol). Pharmacological blockade with L-NAME (LN) resulted in a significant reduction in nitrite production when compared to the groups treated with BaV alone (BaV 0.056 mg/kg_5_min = ±26 µmol; LN + BaV_5_min = ±15.4 µmol) (Figure 4A,B).

At the dose of 0.28 mg/kg, a significant increase in nitrite production was observed only at 15 min after BaV infusion (Control_15_min = ±9 µmol; BaV 0.28 mg/kg_15_min = ±41 µmol). Following pharmacological blockade with L-NAME, administered 60 min prior to BaV infusion in the experimental group, there was a significant reduction (*p* < 0.05) in nitrite production compared to the group treated with BaV alone (Figure 4C).

## 3. Discussion

The results demonstrate that *Bothrops atrox* venom exerts significant effects on renal vascular and functional parameters. The observed reduction in perfusion pressure and vascular resistance highlights the hemodynamic impact of the venom, which may be partially mediated by the release of vasoactive substances.

The elevation in glomerular filtration rate and urinary flow, particularly at later time points, suggests a biphasic response to venom exposure, possibly involving compensatory mechanisms or direct tubular effects. Alterations in electrolyte transport, especially in sodium and chloride handling, further support the idea of tubular dysfunction induced by the venom.

The neuropeptide bradykinin was first identified and described as a key modulator of blood pressure by Rocha e Silva and colleagues in 1949. Subsequent research revealed that *Bothrops jararaca* venom contains significant quantities of bradykinin-potentiating peptides (BPPs), which act as natural inhibitors of angiotensin-converting enzyme (ACE) [15,19,20]. A recent study identified 126 novel peptides in the venom of *Lachesis muta*, with BPPs being the most abundant, reinforcing their therapeutic potential in blood pressure modulation and the development of new antihypertensive agents [8].

Histological analysis confirmed discrete morphological changes in BaV-perfused kidneys, characterized by proteinaceous deposits in renal tubules, indicating possible glomerular or tubular damage. This finding aligns with the functional data and suggests structural consequences of venom exposure.

The significant increase in nitrite production following BaV administration, and its reversal by L-NAME, implicates nitric oxide (NO) in the renal effects of the venom. NO is known to be involved in vasodilation and modulation of renal hemodynamics, and its overproduction may contribute to the observed alterations.

Together, these findings provide new insights into the renal pathophysiology associated with *Bothrops atrox* envenomation and support the involvement of NO as a mediator of venom-induced effects.

Additionally, the present findings have translational relevance, particularly in regions where *Bothrops atrox* envenomation is highly prevalent and represents a major public health concern. Acute kidney injury is a severe and sometimes fatal complication of snakebite envenoming, and understanding the early renal effects induced by specific venom components may contribute to the development of more effective therapeutic strategies.

From a pharmacological perspective, the use of isolated perfused kidney models offers a controlled platform to dissect the renal actions of complex biological toxins, independent of systemic variables. This approach is particularly valuable in snake venom research, where systemic inflammatory responses and hemodynamic instability often confound in vivo findings.

Moreover, our results reinforce the importance of early intervention in envenomation cases, especially considering that some venom-induced renal effects may occur rapidly and independently of systemic hypotension. Future studies should explore whether specific inhibitors—such as metalloproteinase blockers or nitric oxide synthase inhibitors—might attenuate the renal damage observed here.

It is also worth noting that the elevation of nitric oxide, while potentially protective in the short term due to its vasodilatory properties, may paradoxically exacerbate cellular damage through nitrosative stress. This dual role of NO highlights the complexity of its involvement in venom-induced pathology and underscores the need for detailed mechanistic studies.

Finally, the integration of morphological, functional, and biochemical parameters in this study strengthens the overall conclusion that *Bothrops atrox* venom exerts direct and multifaceted nephrotoxic effects. These insights can inform both clinical management and the design of adjunct therapies aimed at minimizing organ damage in snakebite victims.

In clinical practice, specific treatment with antivenom remains the cornerstone of snakebite management. However, it is important to consider that antivenom therapy itself can influence renal physiology. Fab antivenoms, due to their low molecular weight, can easily reach extravascular compartments and are predominantly eliminated via the kidneys, with an average half-life ranging from 2 to 24 h. This pharmacokinetic profile may contribute to renal stress or toxicity, particularly in patients with preexisting kidney dysfunction or those exposed to nephrotoxic venom components. These aspects should be carefully monitored during antivenom administration and underscore the need for integrated therapeutic strategies in envenomation cases [21].

Recent investigations have emphasized the nephrotoxic potential of *Bothrops* venoms, particularly *B. atrox*, which may trigger acute kidney injury (AKI) through mechanisms involving direct tubular injury, inflammatory responses, and disturbances in coagulation pathways. Proteomic analyses of urine from *B. atrox*-envenomed patients revealed the presence of biomarkers linked to AKI, including acute-phase and inflammatory proteins, thus reinforcing the clinical relevance of venom-induced renal impairment [22]. Additionally, experimental studies with *Bothrops jararaca* venom demonstrated significant renal dysfunction in rats, including decreased glomerular filtration rate, altered sodium reabsorption, and histopathological evidence of tubular damage [23]. These outcomes highlight the central role of venom constituents such as metalloproteinases and phospholipases A_2_ in the pathogenesis of venom-induced nephrotoxicity.

In the present study, the elevation in nitrite levels observed following BaV administration supports the involvement of nitric oxide (NO) in venom-induced renal effects. The increase in NO production may contribute to vasodilation and enhanced vascular permeability, thereby aggravating renal dysfunction. These findings are consistent with prior evidence indicating that NO plays a dual role in modulating renal hemodynamics and promoting oxidative stress during snake envenomation [23].

## 4. Conclusions

Renal alterations induced by *Bothrops atrox* venom (BaV) were observed in nearly all renal parameters evaluated in this study. The venom demonstrated a capacity to reduce renal vascular parameters, indicating vasoactive properties; increase sodium excretion; and promote mild histological changes in perfused kidneys. Regarding the involvement of nitric oxide (NO) in BaV-induced effects, a significant increase in nitrite production was observed, indicating elevated NO levels during *Bothrops atrox* envenomation. Therefore, this mediator may play a role in the renal effects caused by the venom.

From a translational and clinical perspective, these results contribute to a better understanding of the pathophysiological mechanisms underlying snakebite-induced acute kidney injury, a major complication in envenomation cases. The use of an isolated perfused kidney model provides a valuable tool to dissect early renal responses to venom exposure and may aid in the identification of therapeutic targets to prevent or mitigate organ damage.

Future studies should aim to identify the specific venom components responsible for the observed renal effects and explore the molecular pathways involved in nitric oxide-mediated toxicity. In addition, evaluating the protective potential of pharmacological inhibitors may offer new insights for the development of adjunctive therapies in snakebite treatment.

## 5. Materials and Methods

*Bothrops atrox* (BaV) venom was generously supplied by Professor Dr. Diva Maria Borges Nojosa, who serves as the coordinator of the Regional Center for Ophidology (NUROF) of the Department of Biology at the Federal University of Ceará. The drugs and salts used in the dissertation were obtained from Sigma (Burlington, MA, USA).

All experimental procedures involving animals were conducted in compliance with established ethical standards, including the U.K. Animals (Scientific Procedures) Act of 1986, the EU Directive 2010/63/EU on the protection of animals used for scientific purposes, and the guidelines outlined in the NIH Guide for the Care and Use of Laboratory Animals (NIH Publication No. 8023, revised, 1978). This study was approved by the Ethics Committee on Animal Research of Universidade Federal do ceará (CEUA/UFC), authorization Number: 55/09.

To assess renal responses without the influence of systemic variables, isolated kidney perfusion was employed. In summary, adult male Wistar rats (weighing between 260 and 320 g) were subjected to 24 h of fasting with unrestricted access to water. Anesthesia was induced using sodium pentobarbital (50 mg/kg, intraperitoneally), after which the right kidney was surgically exposed. Cannulation of the right renal artery was performed through the mesenteric artery, preserving uninterrupted blood flow, following the method established by Bowman (1970) [24].

The perfusion medium consisted of a modified Krebs–Henseleit solution (MKHS) containing 114.00 mmol/L of NaCl, 4.96 mmol/L of KCl, 1.24 mmol/L of KH_2_PO_4_, 0.5 mmol/L of MgSO_4_·7H_2_O, 2.10 mmol/L of CaCl_2_, and 24.99 mmol/L of NaHCO_3_. To this base, bovine serum albumin (6% BSA, fraction V), urea (0.075 g), inulin (0.075 g), and glucose (0.15 g) were added, yielding a total volume of 100 mL. The pH of the solution was adjusted to 7.4. During each experiment, 100 mL of MKHS was recirculated for a period of 120 min.

Perfusion pressure (PP) was recorded directly at the distal end of the stainless steel cannula positioned within the renal artery. Urine and perfusate samples were collected at 10-min intervals for analysis of sodium, potassium, and chloride levels by ion-selective electrodes (RapidChem 744, Bayer Diagnostic, Reading, UK); and inulin, as described by Walser et al. (1955) [25] with modifications introduced by Fonteles et al. (1983) [26]. Osmolality was determined using a vapor pressure osmometer (Wescor 5100C, Pittsburgh, PA, USA). The TSP (1 μg/mL, 3 μg/mL and 4.5 μg/mL) was introduced into the perfusion system 30 min after initiation of each procedure. The following parameters were evaluated: perfusion pressure (PP), renal vascular resistance (RVR), urinary flow (UF), glomerular filtration rate (GFR), and the tubular transport percentages of sodium (%TNa^+^), potassium (%TK^+^), and chloride (%TCl), according to the method described by Martinez-Maldonado and Opava-Stitzer (1978) [27]. These measurements were compared to the internal control group, based on data collected during the initial 30 min of perfusion (n = 6).

For histological evaluation, both kidneys (right and left) were fixed in formaldehyde and subsequently processed. Tissue fragments underwent dehydration, clearing, and sectioning into 3 µm slices. Hematoxylin-eosin (HE) staining was applied, and the samples were examined under a light microscope.

Nitric oxide (NO) production was assessed using male Wistar rats, which were anesthetized with halothane prior to the intrapenile administration of *Bothrops atrox* venom at doses of 0.056 mg/kg, 0.168 mg/kg, or 0.28 mg/kg. Animals were assigned to experimental groups (n = 4 per group). Blood samples were collected from the orbital plexus at two time points—5 and 15 min post-venom administration—prior to euthanasia. Samples were centrifuged, and the supernatant was stored at −70 °C for subsequent nitrite quantification.Pharmacological inhibition of NO synthesis was performed in additional experimental groups (n = 4) by intraperitoneal administration of L-NAME (10 mg/kg) 60 min before the venom injection at the same doses. NO production was indirectly determined by measuring total nitrite (NO_2_^−^) levels using the Griess reaction and analyzed spectrophotometrically. Measurements were conducted in 96-well microplates and performed in duplicate. Fifty microliters of each experimental sample was added per well. A standard curve for nitrite (NO_2_^−^) quantification was generated using a series of dilutions (80, 40, 20, 10, 5, 2.5, 1.25, and 0.625 μmol), with 50 μL from each concentration dispensed in duplicate into a separate microplate. Subsequently, 50 μL of sulfanilamide solution was added to each well, and the plate was incubated for 10 min at room temperature, protected from light. Following this step, 50 μL of N-(1-naphthyl)ethylenediamine dihydrochloride solution was introduced, and a second incubation was carried out under the same conditions [17]. The formation of a purple/magenta color was then assessed immediately using a microplate spectrophotometer at a wavelength range of 520–550 nm. Absorbance values of the experimental samples were compared with the standard curve to calculate nitrite concentrations.

Data are presented as mean ± standard error of the mean (SEM), based on five independent experiments conducted for each group. Statistical analysis was performed using Student’s *t*-test and one-way ANOVA, followed by the Newman–Keuls post hoc test when appropriate. Differences were considered statistically significant when *p* < 0.05.

## Data Availability

The original contributions presented in this study are included in this article. Further inquiries can be directed to the corresponding author.
